# Preliminary Study of Sars-Cov-2 Occurrence in Wastewater in the Czech Republic

**DOI:** 10.3390/ijerph17155508

**Published:** 2020-07-30

**Authors:** Hana Mlejnkova, Katerina Sovova, Petra Vasickova, Vera Ocenaskova, Lucie Jasikova, Eva Juranova

**Affiliations:** 1 T. G. Masaryk Water Research Institute, Public Research Institution, Podbabska 2582/30, 160 00 Prague, Czech Republic; hana.mlejnkova@vuv.cz (H.M.); vera.ocenaskova@vuv.cz (V.O.); lucie.jasikova@vuv.cz (L.J.); eva.juranova@vuv.cz (E.J.); 2 T. G. Masaryk Water Research Institute, Public Research Institution, Brno Branch, Mojmirovo namesti 16, 612 00 Brno, Czech Republic; 3 Veterinary Research Institute, Public Research Institution, Hudcova 296/70, 621 00 Brno, Czech Republic; vasickova@vri.cz

**Keywords:** wastewater-based epidemiology, early warning system, virus, coronavirus, SARS-CoV-2, wastewater, epidemic, RT-qPCR

## Abstract

The virus SARS-CoV-2, which has caused the recent COVID-19 pandemic, may be present in the stools of COVID-19 patients. Therefore, we aimed to detect SARS-CoV-2 in wastewater for surveillance of SARS-CoV-2 in the population. Samples of untreated wastewater were collected from 33 wastewater treatment plants (WWTPs) of different sizes within the Czech Republic. SARS-CoV-2 RNA was concentrated from wastewater and viral RNA was determined using real-time reverse transcription polymerase chain reaction (RT-qPCR). SARS-CoV-2 RNA was detected in 11.6% of samples and more than 27.3% of WWTPs; in some of them, SARS-CoV-2 was detected repeatedly. Our preliminary results indicate that an epidemiology approach that focuses on the determination of SARS-CoV-2 in wastewater could be suitable for SARS-CoV-2 surveillance in the population.

## 1. Introduction

Since the beginning of the year 2020, there has been significantly increased awareness of the existence of microorganisms that cause disease. One such representative group of viruses is SARS-CoV-2. This virus was able to spread a respiratory disease called COVID-19 to the level of a global pandemic in a very short period of time.

In the Czech Republic, the first case of COVID-19 was documented on 1 March 2020. The maximum number of concurrent cases to date of the epidemic was reached in the middle of April (11 April 2020) with 4651 COVID-19 infected persons. The overall number of infected persons at the end of this study in the middle of June (14 June 2020) was 10,064 (data provided by the Ministry of Health of the Czech Republic).

Coronaviruses, whose name was derived from their crown-like shape (*corona* in Latin), are enveloped, positive-sense RNA viruses. The genome of coronaviruses is encoded in a single strand of RNA with a positive polarity of approximately 30,000 bases, making these viruses the largest known RNA viruses with a non-segmented genome. Coronaviruses are known to cause respiratory and gastro-intestinal infections in animals and humans [1,2]. They are usually responsible for the common cold or mild respiratory infections; nevertheless, severe infections such as Severe Acute Respiratory Syndrome (SARS), Middle East Respiratory Syndrome (MERS), and most recently COVID-19 have also been described [3,4,5,6,7,8].

Coronaviruses are mainly transmitted through respiratory secretions. However, the RNA of SARS, MERS and even SARS-CoV-2 has been detected in stool and urine samples from infected persons [9,10,11,12,13,14,15,16,17,18]. Fecal excretion of the virus was found in approximately 50% of infected persons [9,10,11,12,13,14,15,16,17,18]. SARS-CoV-2 RNA has also been detected in the feces of COVID-19 patients exhibiting only mild or no symptoms, even long after they resolved and tested negative for SARS-CoV-2 RNA in respiratory secretions. Thus, viral shedding can last longer than shedding from the respiratory tract [9,19,20]. However, it is still unclear whether SARS-CoV-2 can be transmitted via the fecal–oral route.

The sources of SARS-CoV-2 in wastewater are mainly feces, respiratory secretions, contaminated personnel products (e.g., wet wipes, diapers, paper tissues), and urine [20,21,22,23,24,25,26,27]. The obtained data indicate that the number of viruses excreted in wastewater increases along with the number of infected persons in the population. The occurrence of SARS-CoV-2 RNA in wastewater has been described in several recent studies, for example from the Netherlands, Italy, USA, France, Spain, Australia, and Pakistan [24,28,29,30,31,32,33,34]. Research teams found SARS-CoV-2 RNA in wastewater soon after the epidemic occurred [24]. However, the most recent observation from Italy revealed that SARS-CoV-2 RNA was present in wastewater samples in December 2019, long before the country’s first confirmed cases [35].

The viral load in feces and the detection limit of analyses are essential limiting factors for the success of SARS-CoV-2 monitoring in wastewater. Hata et al. [29] suggested that the limit of SARS-CoV-2 RNA detection in wastewater is roughly estimated as 2 copies/mL using RT-qPCR, meaning that 6.0 × 10^10^ copies per 100,000 inhabitants must be present in the sewer catchment area for the detection of SARS-CoV-2 RNA in wastewater when the daily wastewater quantity is 300 L/person/day [29].

Wastewater monitoring has been successfully used for tracking drug consumption, pharmaceutical use, water pollution, and antibiotic resistance [36,37,38]. However, through SARS-CoV-2 detection in wastewater, it is also possible to obtain unique epidemiological information on its occurrence in the population (presence, absence, and trends: increase, stagnation, decrease) [39,40,41,42,43,44]. A properly set up wastewater monitoring system enables outbreak monitoring and trend observations in the numbers of viral diseases of future periods and may be used as an early warning tool to set up a system for effective surveillance of disease spread.

In this study, we aimed to detect SARS-CoV-2 RNA in untreated wastewater samples in the Czech Republic. Observed data indicated the applicability of a wastewater monitoring approach for SARS-CoV-2 surveillance in the population.

## 2. Materials and Methods

### 2.1. Wastewater Sampling

From April to June 2020, untreated wastewater samples were collected weekly (weeks 17–25 in 2020) from inflow of 33 wastewater treatment plants (WWTPs) within the Czech Republic. WWTPs were chosen preferably in regions with higher prevalence of COVID-19. An overview of the selected WWTPs according to the population served is given in Table 1. WWTP catchments represented approximately 12.2% of the population of the Czech Republic.

In total, 112 samples were taken by WWTP operators. Most samples were 24 h composite samples (time or flow dependent) of 500 mL and were collected from WWTP inflows. Immediately after sampling, samples were cooled and stored at 5 ± 3 °C until analyzed. The analysis was performed within 48 h after sampling.

### 2.2. Sample Concentration and RNA Extraction

The wastewater concentration was conducted by direct flocculation [45] using beef extract solution (3.0% w/v, pH 9.5; Sigma-Aldrich, St. Louis, USA) in glycine buffer (0.05 M). Briefly, 500 mL of the wastewater sample was acidified (pH 3.5 ± 0.1) and 10 mL of beef extract flocculated by the addition of 1 and 0.1 M HCl (pH 3.5 to 3.0; a visible floc formed) was added. The suspension was stirred for 10 h to allow the viruses present to adsorb to the flocs and subsequently centrifuged at 10,000× *g* for 30 min at 4 °C. The pellet was dissolved in 8 mL of phosphate-buffered saline (PBS) and RNA isolated with the NucliSENS^®^ miniMAG^®^ system (BioMérieux, Marcy l’Etoile, France) according to the manufacturer’s instructions.

### 2.3. Process Control Virus

According to ISO 15216-2:2019 [46], the virus selected for use as a process control should provide similar morphological and physicochemical quality to the target virus and should be sufficiently distinct genetically that the detecting assay does not cross-react. Therefore, transmissible gastroenteritis coronavirus (TGEV, strain M42, Collection of zoopathogenic organisms, Veterinary Research Institute, p.r.i., Brno, the Czech Republic) from infected pigs was used as a process control virus (PCV) for the purposes of SARS-CoV-2 RNA detection in wastewater samples. Prior to any sample treatment, each sample was artificially contaminated with 5 µL of TGEV (106 genome equivalents (GE)/µL) to verify the isolation of viral RNA. TGEV-specific RNA was detected using primers and probes adopted from the literature [47]. An internal amplification control was incorporated into the RT-qPCR assay [48].

### 2.4. RT-qPCR Analysis

The presence of SARS-CoV-2 RNA in wastewater was detected by real-time reverse transcription polymerase chain reaction (RT-qPCR) using EliGene COVID19 Basic A RT kit (Elizabeth Pharmacon, Brno, the Czech Republic) according to the manufacturer’s instructions. The kit is applicable to RNA isolated from all relevant clinical specimens (nasopharyngeal swabs, saliva, sputum, serum, plasma, and feces), as well as to environmental samples (wastewater, etc.). Specificity of the assay targeting three parts of the SARS-CoV-2 genome is declared by the manufacturer. Each sample of isolated RNA was tested by RT-qPCR in duplicate. To reveal possible inhibition of the assay and thus avoid false negative results, undiluted and 10× diluted samples of extracted RNA were analyzed. RT-qPCR assays targeting SARS-CoV-2, as well as the TGEV genome, included internal amplification controls. Samples with a cycle quantification value (Cq) < 40 were considered positive. Amplification and fluorescence detection were performed on a LightCycler 480 (Roche Molecular Diagnostics, Mannheim, Germany). The subsequent analysis was carried out using the “Fitpoint analysis” of the LightCycler 480 Software release 1.5.0 (version 1.5.0.39; Roche Molecular Diagnostics, Mannheim, Germany). The absolute quantification of TGEV (PCV, GE/500 mL) was completed on the basis of a calibration curve derived from a 10-fold diluted in vitro transcript [48]. Detection and quantification of TGEV (PCV), and calculation of processing efficiency was performed as previously described [49]. Samples with a processing efficiency < 1.0% were not considered for further analysis.

## 3. Results

**Figure 1:**
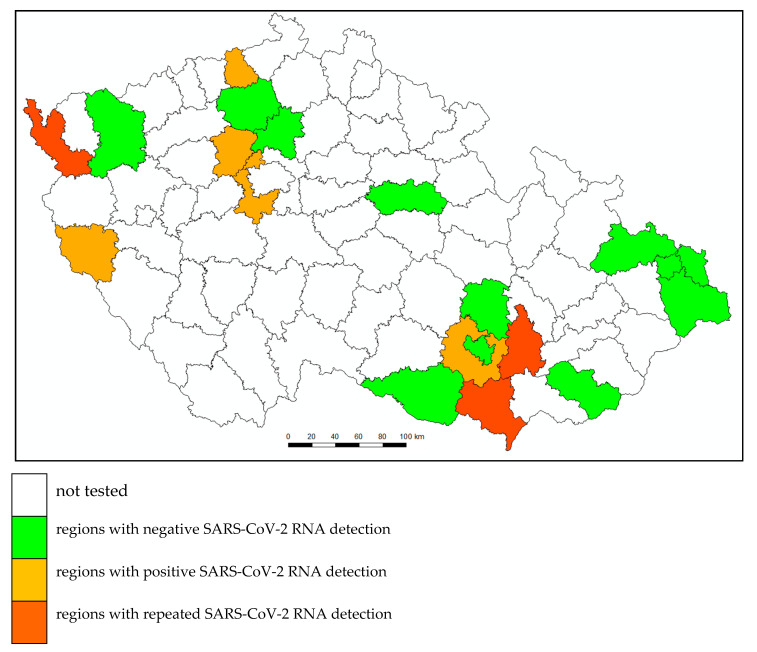
Regions of the Czech Republic where SARS-CoV-2 RNA was detected in wastewater.

In this study, wastewater samples from 33 WWTPs were collected and subjected to SARS-CoV-2 RNA detection. Out of all analyzed samples (112), 13 (11.6%) were found to be positive. SARS-CoV-2 RNA positive samples were found at nine WWTPs. SARS-CoV-2 RNA-positive samples from three WWTPs were found repeatedly (twice and three times, respectively). Regions of the Czech Republic where SARS-CoV-2 RNA was detected in wastewater are identified in Figure 1. Prevalence of COVID-19 cases in those regions varied between 24 and 561 cases per 100,000 inhabitants (based on RT-qPCR detection of SARS-CoV-2 from respiratory secretions provided by authorized laboratories; data provided by the Ministry of Health of the Czech Republic).

Based on the detection and quantification of TGEV (PCV), the mean efficiency of whole wastewater sample analysis was 35.53% with a standard deviation of 13.04. Cq ranging between 37 and 40 was achieved for seven (53.8%) influent wastewater samples, a lower Cq of 34–37 was also observed (six samples; 46.2%).

Table 2 shows a summary of the occurrence of positive wastewater samples and Cq.

## 4. Discussion

In this study, we aimed to detect SARS-CoV-2 in wastewater to use this approach for the surveillance of SARS-CoV-2 in the population.

For this purpose, a procedure based on direct flocculation [45] with a bovine extract solution was used to concentrate the viruses in wastewater, and an RT-qPCR assay was employed to reveal the possible presence of SARS-CoV-2 RNA. Losses of target viruses can occur at all steps during sample processing. Therefore, each step of the analytical procedure should be monitored, and the level of virus recovery should be determined for each sample [46]. TGEV as a PCV was used and the level of process efficiency was consequently specified in each analyzed sample (mean 35.53% with a standard deviation of 13.04). Water samples contain substances that may inhibit RT-qPCR. To control the RT-qPCR of an individual sample, an RNA internal amplification control should be used to reveal inhibition of the assay and thus avoid false negative results. Therefore, a commercially available kit (EliGene COVID19 Basic A RT; Elizabeth Pharmacon, Brno, the Czech Republic) containing such a control was applied. To date, other methods using two-phase separation (PEG-dextran method; designed for poliovirus detection) or ultracentrifugation associated with WHO-recommended or “homemade” RT-qPCR have been reported [24,30]. Regardless, the present results provide evidence of the suitability and validity of the described procedure for wastewater analysis.

A total of 112 wastewater samples from WWTPs from 20 regions (out of 76) in the Czech Republic were analyzed and thus a large area was inspected. A positive signal was observed for only 11.6% of samples of untreated wastewater. The percentage is lower than expected. The mild course of the epidemic in the Czech Republic and decline in the number of infected inhabitants during the study may be reasons why such numbers were observed. Research in other countries (e.g., Spain, France, and the Netherlands) reported the detection of SARS-CoV-2 RNA, often with higher number of positive samples [24,28,29,30,31,32,33,34]. Positive samples were found even in areas with low prevalence of COVID-19 infections as described by Randazzo et al. [32]. For some of the WWTPs, there was only positive detection in one of the nine weeks analyzed even if the infectious process usually lasts more than one week. Several explanations of this finding can be mentioned: COVID-19-infected persons registered according to their permanent residence and cured at the other places, the occurrence of asymptomatic cases of COVID-19 with the virus excretion, inconsistent excretion of the virus with feces of COVID-19 patients, or different amounts of wastewater on the date of sampling.

Virus shedding in feces was confirmed by other studies [9,10,11,12,13,14,15,16,17,18] but it is still unclear whether the viral particles in wastewater are vital and infective. According to previous studies [50,51], the vitality of SARS-CoV-2 in wastewater does not seem to be significant. SARS-CoV-2 transmission via wastewater has not been observed, probably due to its poor stability in wastewater and sensitivity to disinfectants [8,9].

Wastewater monitoring of SARS-CoV-2 seems to be usable in wastewater-based epidemiology as a good monitoring and surveillance system for COVID-19 in the population. This approach can provide rapid and reliable information about the disease outbreaks and their trends in the population. It seems to be suitable for surveillance of mild, subclinical, or asymptomatic cases [25,40,41,42,43,44]. Our preliminary results confirmed the potential of this approach for use as an early warning tool for effective surveillance of the diseases spread. In the future, correlation of our results with the exact number of infected people in the monitored areas (i.e., delicate data from public health sector), wastewater dilution, and other factors (e.g., network size, rainfall) will be performed. After this evaluation, the applicability of this approach for the early warning system as a suitable complementary method to clinical testing will be assessed.

## 5. Conclusions

Our results confirmed the assumption of the presence of SARS-CoV-2 RNA in wastewater from the inflow of WWTPs, however, not to the expected extent. A final evaluation and interpretation of results will be performed after the correlation of all accessible data. The preliminary results of wastewater monitoring seem to be applicable as an early warning system for COVID-19 surveillance in the population.

## Figures and Tables

**Table 1:** The overview of the selected WWTPs according to the population served.

WWTP Capacity According to the Population Served (Number of Inhabitants)	Number of WWTPs Involved in the Study
<2000	2
2000–10,000	7
10,000–100,000	21
>100,000	3

**Table 2:** Results of SARS-CoV-2 RNA detection in wastewater samples obtained by EliGene COVID19 Basic A RT kit (Elizabeth Pharmacon, Brno, the Czech Republic). The results include processing efficiency calculated for each sample analyzed (%).

WWTP Code	Size of WWTP	Week 17	Week 18	Week 19	Week 20	Week 21	Week 22	Week 23	Week 24	Week 25
B1	L	−28.06%	−23.52%	−33.95%	−39.59%	−33.97%	−31.27%	−32.76%		
B2	M	−26.25%	+(36.53) * 25.50%	+(36.36) * 25.28%	−35.51%	−42.95%	+(38.77) 36.75%	−39.79%	−39.48%	−43.51%
B3	M		−43.22%	−46.72%						
B4	M		−46.92%	−33.07%						
B5	S			+(39.62) 27.75%	+(34.40) * 24.17%	−33.10%	−42.23%	−31.58%		
C1	S			−31.29%	−24.16%					
C2-A	M						−41.38%	−43.15%		
C2-B							−47.95%	−40.27%		
C2-E							−46.90%	−23.06%		
C2-F							−30.12%	−47.29%		
D1	S		−32.20%	−26.92%						
D2	M					−29.49%	+(38.84) * 23.27%	−29.82%		
F	M				−28.20%			−37.01%		
H1	S	−44.76%	+(37.19) 37.09%	−39.44%	−46.59%	−31.95%	−26.63%	−26.36%		
I	M	−48.57%	−42.40%	−28.96%						
K	M							−45.84%		−46.50%
K1	M	−30.91%	−39.92%	−27.36%	−34.46%	−44.94%	−23.71%	−46.84%		
L1	M			−26.63%	−31.48%	−22.90%		−31.30%		
M1	M				+(36.59) 42.86%	−46.01%	+(39.35) 33.53%	−31.82%		
M2	M						−41.92%			
N1	XS		−37.20%	−24.92%						
O	M				−36.78%			−25.42%		
OV	L							−29.64%		−26.10%
P1	XS	−35.13%	+(34.65) 44.05%	−39.52%						
P2-A	L				−30.25%			−34.68%		
P2-B					−44.59%					
R1	S	−29.08%	−26.92%	−22.49%						
S	S			+(38.55) 33.40%	−46.83%	−43.27%	−25.95%	−44.14%		
T	M	−23.45%	+(34.97) 36.93%	−30.50%	−33.10%	−39.95%				
T1	M	−24.85%	−40.09%	−44.82%						
U	M				−46.58%	−46.69%				
U1	M					−47.82%		−46.59%		
UB	M				−35.75%	−36.88%				
V	M			−34.75%						
V1	M						+(39.86) 46.68%	−45.51%		
Z	S	−28.77%	−25.07%	−28.91%						
Z1	M		−40.34%	−44.84%						

Size of WWTP according to population served: XS, <2000; S, 2000–10,000; M, 10,000–100,000; L, >100,000; +, positive SARS-CoV-2 RNA detection; −, negative SARS-CoV-2 RNA detection; * results originating from 10-fold diluted isolated RNA; Cq (cycle quantification value) is in brackets.
